# Maintenance Therapy with Nebulizers in Patients with Stable COPD: Need for Reevaluation

**DOI:** 10.1007/s41030-020-00120-x

**Published:** 2020-05-20

**Authors:** Paul D. Terry, Rajiv Dhand

**Affiliations:** Department of Medicine, Graduate School of Medicine, University of Tennessee Medical Center, Knoxville, TN USA

**Keywords:** COPD, Inhalation device, Inhalers, Lung function, Nebulizers, Patient preference, Patient-reported outcomes, Treatment

## Abstract

Patients with stable COPD rely heavily on inhaled bronchodilators and corticosteroids to control symptoms, maximize quality of life, and avoid exacerbations and costly hospitalizations. These drugs are typically delivered by hand-held inhalers or nebulizers. The majority of patients are prescribed inhalers due to their perceived convenience, portability, and lower cost, relative to nebulizers. Unfortunately, poor inhaler technique compromises symptom relief in most of these patients. In contrast to one or two puffs through an inhaler, nebulizers deliver a drug over many breaths, through tidal breathing, and hence are more forgiving to poor inhalation technique. To what extent susceptibility to errors in their use may influence the relative effectiveness of these two types of inhalation device has received little attention in COPD research. In 2005, a systematic review of the literature concluded that nebulizers and inhalers are equally effective in patients who are adequately trained to use their inhalation device. This conclusion was based on two small clinical trials that only examined objective measures of lung function. Since then, additional studies have found that maintenance therapy administered by nebulizers could improve patients’ reported feelings of symptom relief, quality of life, and satisfaction with treatment, compared to therapy administered by inhalers. Because it has been 15 years since the publication of the systematic review, in this article we summarize the results of studies that compared the effectiveness of inhalers with that of nebulizers in patients with stable COPD and discuss their implications for clinical practice and need for future research.

## Key Summary Points

**Table:** 

Most patients with stable COPD are prescribed maintenance therapy via an inhaler due to the perceived convenience of inhalers compared to nebulizers; however, poor inhaler technique compromises symptom relief in a high percentage of these patients.
Inhaler use training is thought to mitigate any disadvantage of inhalers regarding their effectiveness in relieving patient symptoms, but few studies have addressed the comparative effectiveness and outcomes of nebulized versus inhaler-based therapy for COPD maintenance.
We conducted a literature search and reviewed consensus group statements and the results of studies that compared the effectiveness of inhalers and nebulizers in patients with stable COPD.
Recent investigations, especially those that include patient perceptions as an outcome measure, do not support the equivalence of bronchodilator therapy with nebulizers and inhalers. Prospective, long-term clinical trials using long-acting bronchodilators, with or without inhaled corticosteroids, are needed to evaluate the role of nebulizers for maintenance therapy in patients with stable COPD.

## Introduction

Chronic obstructive pulmonary disease (COPD) is the fourth leading cause of death in the US, affecting over 15 million Americans [1] and over 300 million individuals worldwide [2]. In a recent editorial, the Global Initiative for Chronic Obstructive Lung Disease (GOLD) Board of Directors drew attention to the importance of COPD as a serious public health problem, its role as one of the most important and preventable causes of global inequalities in health outcomes, and the need for a coordinated campaign to reduce its worldwide impact [3].

Patients with COPD rely heavily on inhaled bronchodilators and corticosteroids (ICS) to control symptoms, maximize quality of life, and avoid exacerbations and costly hospitalizations. Reductions in mortality with inhaled “triple” bronchodilator/ICS combinations have also been suggested [4]. These drugs are typically delivered by inhalers, either pressurized metered-dose inhalers (pMDIs), dry powder inhalers (DPIs), or soft mist inhalers (SMIs), or by nebulizers. Each aerosol delivery device has advantages and disadvantages related to portability, ease and speed of use, and cost [5, 6]. The relative convenience of inhalers led to their widespread acceptance by physicians as the primary mode of inhalation delivery for maintenance therapy in ambulatory settings [7], whereas nebulizers are mainly prescribed for rescue treatment with short-acting bronchodilators for relief of acute dyspnea. Unfortunately, a large majority, up to 94%, of patients with COPD do not receive optimal relief from disabling symptoms because they do not use their inhalers appropriately [8]. Compromised inhaler technique, as well as poor adherence to medication, jeopardize health outcomes and add to the considerable economic burden of COPD, by as much as 5 billion dollars annually, according to one estimate [9].

Inhalers are subject to several errors in their use that may result in inadequate symptom relief for patients [10–12]. Many patients, especially the elderly, have problems coordinating inhalation with actuation of pMDIs—a critical step for efficient aerosol delivery with pMDIs. DPIs do not require such coordination because device actuation is achieved by the patient's inhalation. DPIs do, however, require a fast and forceful inhalation for optimal aerosol generation, and generating the inspiratory flow required for effective function of DPIs can be problematic for many patients with COPD [13–15]. SMIs provide a slower-moving mist, but hand-breath coordination, hand strength, and breath holding are still needed; errors in SMI use remain common [12]. In contrast, nebulizer therapy is provided with tidal breathing, and hence is less subject to poor inhalation technique; a forceful inhalation is not necessary, and coordination is not an issue. Nebulizers convert solutions and suspensions into small droplets, and they are particularly well suited to deliver larger doses of medication than is practical with inhalers. However, nebulizers have a long-standing reputation as being more expensive and time consuming, less portable, and requiring more maintenance, than inhalers [16].

Despite an increasing awareness of the problem and health implications of widespread inhaler misuse [10–12], not enough is known regarding the comparative effectiveness and outcomes of nebulized versus inhaler-based therapy for COPD maintenance [17]. Therefore, we review here the results of studies that compared the effectiveness of inhalers and nebulizers in patients with stable COPD and discuss their implications for clinical practice and need for future research.

## Methods

Studies included in this review were identified from previous review articles and consensus reports, and by searching the PubMed (MEDLINE) database for comparative studies using terms such as “COPD,” “nebulizer,” “inhaler,” “lung function,” “patient-reported outcomes,” “FEV_1_,” and by cross-referencing citations in identified studies. We sought to include all studies that compared the effects of regular (maintenance) treatment with inhalers versus nebulizers. Outcome measures of interest included lung function, quality of life, patients’ device preferences, and satisfaction with treatment. We included one study that analyzed data from patients with both COPD and asthma [18]; study results were not stratified by disease type. We excluded studies of hospitalized patients and those of patients experiencing an acute exacerbation of COPD (AECOPD). Our commentary is based on a narrative literature review, without using meta-analysis or other statistical summary of data. This article is based on previously conducted studies and does not contain any studies with human participants or animals performed by either of the authors.

## Consensus Group Statements, Clinical Trials, Observational Studies, and Patient Surveys

### Consensus Group Statements

At least seven consensus groups have considered the literature, and to some extent the prevailing practices and beliefs, related to nebulizers and the choice of aerosol delivery device for maintenance treatment in patients with stable COPD. Here, we briefly summarize consensus group recommendations regarding nebulizer use in maintenance treatment.

#### National Association for Medical Direction of Respiratory Care (NAMDRC)

In 1996, NAMDRC stated, in their Guidelines for the Use of Nebulizers in the Home and at Domiciliary Sites [19], that “the indications for use of nebulizers outside of the hospital have not been clearly defined.” The NAMDRC concluded that “generally, if a medication is available for both pMDI and nebulizer delivery, the desired system is the pMDI, based on its convenience, portability, and cost features.” Exceptions to this recommendation, where home nebulizer use would be indicated, include patients who are incapable of performing the “pMDI maneuver,” those with very low tidal volume, inspired flow, or breath hold capacity, and those who do not benefit sufficiently from pMDI use. At the time of that report, DPIs, especially those delivering long-acting bronchodilators in combination with ICS, were not widely available for maintenance therapy in patients with COPD.

#### British Thoracic Society (BTS) Nebulizer Association of Palliative Care Medicine Project Group

In their 1997 report [20], the BTS stated that although a few patients may benefit from high-dose home bronchodilator treatment delivered by nebulizer, adequate domiciliary bronchodilator treatment for most patients with COPD can be delivered with standard treatment with an inhaler. The BTS panel did not cite any study that compared the two modes of aerosol drug delivery regarding their effectiveness.

#### European Respiratory Society (ERS)

In 2001, an ERS taskforce published guidelines on the use of nebulizers, noting both the shortage of clinical trials of clinical nebulizer use and the poor quality of existing trials [21]. Despite the dearth of quality data, the ERS recommended drug delivery via pMDI for most patients for convenience and simplicity of delivering the lowest effective dose. Only when higher doses of medicine are required, the threshold for which being patient-specific, would nebulizers be recommended.

#### ERS/ISAM Task Force

In 2011, an ERS/International Society for Aerosols in Medicine (ISAM) task force issued a consensus statement regarding what pulmonary specialists should know about new inhalation therapies [22]. The task force noted that, particularly among elderly patients, declines in cognitive function and manual dexterity may compromise the effective use of inhalers, in which case the use of nebulizers was recommended. Otherwise, the use of nebulizers for regular maintenance therapy in patients with COPD was not discussed.

#### American Thoracic Society (ATS)/ERS Statement on Research Questions in COPD

In 2015, an ATS/ERS consensus group published a statement highlighting the types of research that their leading clinicians and researchers believed would have the greatest impact on outcomes in patients with COPD [17]. Based on the paucity of reliable data regarding inhalation device effectiveness, the ATS/ERS recommended “studies that compare outcomes among patients who use an inhaler with those who use a nebulizer.”

#### Indian National Allergy Asthma Bronchitis Institute and Chest Research Foundation (NAABI/CRF)

In 2017, the NAABI/CRF in India issued a consensus document on home nebulization for maintenance treatment of obstructive airway diseases (OADs, i.e., asthma and COPD) [23], citing the lack of any recent international guidelines regarding home nebulization for maintenance treatment of OAD. NAABI/CRF noted, as have others, that some patients, including the elderly, may benefit from nebulizers when inhaler technique is compromised. The NAABI/CRF also noted that advances in nebulizer technology have made them more patient- and pocket-friendly, and that there is increased availability of nebulized drug formulations. However, this panel did not recommend the use of nebulizers for regular maintenance therapy in patients with OAD. Rather, they recommended that every effort should be made to reintroduce drug administration through inhalers to patients currently using nebulizers. The reasons for this recommendation appear to be concern for “the potential for misuse of nebulization in patients with OADs” and an increased risk of adverse effects and acquired infections, although the consensus group cited very limited evidence to support these explanations. In this context, we note that several 1-year studies performed in patients with stable COPD using nebulized formoterol, arformoterol, and revefenacin have not reported a significant increase in serious adverse effects [24–27].

#### Global Initiative on Chronic Obstructive Lung Disease (GOLD)

GOLD is internationally recognized for the development of evidence-based strategy documents, most notably the annual GOLD Reports, for COPD diagnosis, management, and prevention [28]. GOLD’s conclusions regarding regular use of nebulizers for maintenance have evolved since the first GOLD Report was published in 2001 [29]. In that report, and all subsequent reports until 2010, GOLD stated that “Nebulizers are not recommended for regular treatment because they are more expensive and require appropriate maintenance [7].” In 2010, GOLD made cautious recommendations for use of nebulizers: “Many drugs are available as nebulizer solutions and for patients who are severely overinflated and consequently may have very low inspiratory flow rates, there may be theoretical advantages of nebulizers. However, there is little randomized trial evidence for benefit compared to the use of other devices and use of nebulizers will often depend on local preference, availability, and price. Benefit should be judged symptomatically, since changes in lung function may be small and within the limits of repeatability. Nebulized treatment should only be continued if the patients report clear symptomatic benefit that cannot be achieved by simpler, cheaper, and more portable alternatives.” GOLD’s evolution towards accepting nebulizers as a standard inhalation delivery device in patients with stable COPD continued, and the caveats noted above have been absent from the GOLD Reports since 2017. Regarding device effectiveness, GOLD stated recently: “Randomized controlled trials have not identified superiority of one device/formulation. However, patients included in these trials are usually those who master inhalation technique and receive proper education and follow-up regarding this issue, and therefore may not be reflective of normal clinical practice [30].”

#### Summary

Six of these seven consensus groups have expressed a preference for inhalers over nebulizers for regular maintenance therapy for stable COPD, typically for the reasons of cost, convenience, and portability. In their last four annual reports (2017–2020), however, GOLD has not made any blanket recommendation regarding device preference based on those or any other concern. With the exception of three consensus statements by ERS—one alone, followed by statements issued jointly with ISAM and ATS—GOLD is the only organization that has issued a consecutive series of evidence-based strategy documents that contain recommendations related to the choice of aerosol delivery device among patients with stable COPD [29]. Because this series is ongoing, we anticipate that future GOLD reports will continue to provide clinicians an excellent resource for understanding the relative advantages and disadvantages of aerosol delivery devices used for COPD maintenance therapy.

### Clinical Trials

The European Respiratory Society (ERS) noted both the shortage of clinical trials of clinical nebulizer use and the poor quality of existing trials in 2001 [21]. In our view, this remains the case today regarding clinical trials that compared the effectiveness of inhalers versus nebulizers in patients with stable COPD.

#### A Systematic Review of the Literature

In 2005, Dolovich et al. published a systematic review of device selection and outcomes of aerosol therapy [31], which, to date, has been cited over 450 times in the peer-reviewed literature in support of the conclusion that various devices have equivalent effectiveness when they are used appropriately. The authors restricted their review to studies in which the effectiveness of inhalation devices was compared using the same inhaled medicines, thereby eliminating confounding by medication type. Two studies that compared the effectiveness of inhalers versus nebulizers were included in that review [18, 32]. A randomized crossover trial by Hansen et al. [32] found no appreciable or statistically significant difference in lung function between terbutaline delivered by a DPI or nebulizer in 22 patients with severe COPD (Table 1). Another randomized crossover trial by Balzano and coworkers [18] found a 19% greater change in FEV_1_ after treatment with a multidrug combination via nebulizers compared with inhalers among 20 patients (12 with COPD and eight with asthma), a result that was not statistically significant (Table 2). Although patient-reported outcomes were examined by Balzano et al. (see below), this was not the focus of the systematic review. Based on results of these two studies regarding objective measures of lung function, the effectiveness of nebulizers and inhalers was considered by Dolovich et al. to be equivalent in patients adequately trained to use their inhalers.

**Table 1:** Clinical trials of regular treatment with nebulizers versus inhalers and measures of lung function

First author, year	Study type	Sample and comparison	Study findings
Hansen, 1989 [32]	Crossover trial with measurements of outcome once each day, over two consecutive days, up to 60 min after exposure	22 with severe COPD 2 mg terbutaline via DPI vs. 5 mg terbutaline via nebulizer	No appreciable or statistically significant difference in FEV_1_ or FVC was observed according to inhalation device
Ikeda, 1999 [33]	Crossover trial with treatments over seven separate days with effects observed up to 4 h after inhalation	10 with stable COPD 200 mcg and 1000 mcg albuterol via DPI vs. pMDI with a large-volume spacer vs. the same doses via nebulizer	Greater increase in FEV_1_ with inhalers than nebulizers evident with the higher dose of albuterol
Ramlal, 2013 [34]	Crossover trial on 1 day with effects observed 45 min after inhalation	10 with COPD 400 mcg albuterol and 40 mcg ipratropium via pMDI with AeroChamber vs. the same doses via nebulizer	Increase in FEV_1_ was significantly greater via nebulizers than pMDIs with AeroChambers, although results for other parameters of lung function, such as inspiratory capacity and peak inspiratory flow, were statistically similar
Mahler, 2014 [35]	Crossover trial 1 day with effects observed up to 2 h after inhalation	20 with COPD 50 mcg salmeterol dry powder via DPI vs. arformoterol (15 mcg/2 ml) via nebulizer	Volume responses were greater with arformoterol via nebulizer than dry powder salmeterol
Mahler, 2019 [36]	28-day parallel-group clinical trial	206 patients with COPD, including 161 with predicted FEV_1_ < 50%) 175 mcg revefenacin via nebulizer vs. 18 mcg tiotropium dry powder via DPI	Nebulized revefenacin increased trough FEV_1_ in patients with FEV_1_ < 50% predicted and suboptimal peak flow (sPIRF) compared with tiotropium via inhaler

**Table 2:** Clinical trials of nebulizers vs. inhalers that included patient-reported outcomes

First author, year	Study type	Sample and comparison	Study findings
Jenkins, 1987 [39]	8-week crossover trial	19 with stable chronic airflow limitation (number with COPD not stated) Albuterol via pMDI vs. nebulizer	No statistical difference in daily peak expiratory flow (PEFR), severity of symptoms, extra bronchodilator use, or exercise tolerance All patients attributed an improvement in their symptoms to the nebulizers
O’Driscoll, 1992 [40]	Clinical trial of usual inhaler treatment followed by nebulizer treatment	34 with COPD	Approximately half of patients who remained breathless despite receiving bronchodilators delivered by pMDIs or DPIs derived additional benefits from home nebulizer use; the majority of patients with COPD in this study chose to remain on nebulizers for long-term therapy
Balzano, 2000 [18]	2-week crossover trial	12 with COPD, 8 with asthma (combined in analyses) Multidrug combination of 600 vs. 1875 μg of albuterol, 120 vs. 375 μg of ipratropium bromide, and 1000 vs. 3000 μg of flunisolide via pMDI and nebulizer, respectively	A 19% greater change in FEV_1_ after treatment with nebulizers compared with inhalers was not statistically significant The majority (75%) of participants considered treatment more effective with nebulizers than with inhalers
Tashkin, 2007 [41]	12-week randomized clinical trial of patients comparing inhalers, nebulizers, and concomitant therapy	126 with COPD Albuterol plus ipratropium via nebulizer, inhaler, or both	Nebulizers showed better patient-reported outcomes including questionnaire symptoms and quality of life; peak flow and FEV_1_ showed no significant differences; concomitant therapy was better than either alone
Brophy, 2008 [42]	Crossover trial	25 with COPD 120 mcg ipratropium bromide and 600 mcg of albuterol via pMDI with spacer vs. 500 mcg ipratropium bromide and 2.5 mg albuterol via nebulizer	No statistical difference in measures of lung function, 6-min walk distance, breathlessness score, or qualify of life score 60% of patients reported a preference for nebulizers

#### Additional Clinical Trials of Lung Function that did not Include Patient-reported Outcomes

We identified four additional trials that did not consider patient-reported outcomes, including three crossover trials [33–35] and one parallel group trial [36] (Table 1).

In a very small crossover trial in Japan (1999; *n* = 10 with stable COPD), Ikeda et al. found that albuterol delivered via DPI or a pMDI with a large-volume spacer resulted in greater increases in FEV_1_ than via nebulizer [33].

In contrast, a crossover trial by Ramlal et al. ([34], *n* = 41 with COPD) found that increase in FEV_1_ with albuterol and ipratropium bromide was significantly greater via nebulizers than pMDIs with AeroChambers, although results for other parameters of lung function, such as inspiratory capacity and peak inspiratory flow, were statistically similar.

Mahler et al. ([35]; *n* = 20 patients with COPD) found that volume responses were greater with arformoterol via nebulizer than dry powder salmeterol in a relatively small crossover trial. The effectiveness of inhalation devices was compared using different inhaled medicines and, therefore, confounding by medication type is possible.

In a 28-day parallel-group clinical trial, Mahler et al. ([36]; *n* = 206 patients with COPD, including 161 with predicted FEV_1_ < 50%) found that nebulized revefenacin increased trough FEV_1_ in patients with FEV_1_ < 50% predicted and suboptimal peak flow (sPIFR) compared with tiotropium via inhaler. As with the earlier trial by Mahler (discussed above), the effectiveness of inhalation devices was compared using different inhaled medicines.

#### Summary

The large majority of studies reviewed by Dolovich et al. in 2005, upon which the authors based their overall conclusion of aerosol delivery device equivalence, did not perform any comparison between inhalers and nebulizers in patients with stable COPD. The two studies cited in that review that did address the inhaler–nebulizer comparison in patients with stable COPD examined short-term effects of short-acting drugs over short study periods, with small sample sizes and an unknown influence on results in one study from patients with asthma; in other words, the two studies are methodologically very weak. The four additional trials reviewed in this section are weak in similar ways, and two compared different drugs in the two devices. These limitations may explain the conflicting results of those studies, which alternatively showed nebulizer–inhaler equivalence, nebulizer superiority, or nebulizer inferiority. Participants in those clinical trials also tended to receive atypical training to use their prescribed inhalers until they were able to show proficiency, in contrast with “real-life” situations where most patients with COPD do not use inhalers appropriately, due to inadequate training or physical/cognitive limitations [8, 10, 37, 38]. Moreover, those studies tended to focus on objective measures of lung function, which do not correlate well with patient-reported dyspnea symptoms or quality-of-life measures [30]. These limitations warrant caution when applying the overall conclusions of Dolovich et al. specifically to inhaler–nebulizer equivalence. Clearly, there is a need for well-designed comparative efficacy and safety trials with the most effective LABA/LAMA combination, with or without ICS, administered by inhalers versus nebulizers.

#### Clinical Trials of Lung Function that Included Patient-reported Outcomes

The review by Dolovich et al. did not consider patient-reported outcomes when comparing the effects of inhalers and nebulizers [31]. However, the crossover trial of Balzano et al. [18], whose results regarding FEV_1_ provided a basis for Dolovich et al.’ conclusions, found that the majority (75%) of participants considered treatment with the same combination of drugs more effective with nebulizers than with inhalers, even while considering nebulizers to be less convenient. Several additional trials that compared patient-reported outcomes of bronchodilator therapy, mostly using the same drugs via nebulizer versus inhaler, have shown similar results (Table 2).

Jenkins et al. ([39]; *n* = 19 with stable chronic airflow limitation) found albuterol via nebulizer and inhaler statistically equivalent for daily peak expiratory flow, severity of symptoms, extra bronchodilator use, and exercise tolerance, using a crossover trial design. Nonetheless, the authors stated: “All patients reported an improvement in their symptoms during the study period and attributed this to the nebulizers,” notwithstanding the observed statistical equivalence of inhalers and nebulizers regarding lung function.

O’Driscoll et al. ([40]; *n* = 32 with COPD) conducted a trial wherein 2-week treatment with patients’ usual inhalers (without spacers) was followed by a 2-week treatment with pMDI and spacers, followed by an extended period on home nebulizer treatment, forgoing the more typical randomization of treatment order. Patients on nebulizers could choose among several treatment drugs, so medication type was not controlled in analyses. Patients had a mean 20% reduction in their perceived level of breathlessness while using nebulized treatment compared with their usual inhaler therapy. Approximately 50% of patients who had residual symptoms after treatment with inhalers received additional benefits from home nebulizer use. At the study’s closure, most patients chose to continue using nebulizers for regular maintenance therapy rather than revert to inhalers.

Tashkin et al. ([41]; *n* = 126 with COPD) conducted a 12-week randomized clinical trial of patients comparing the effects of albuterol and ipratropium via inhalers, nebulizers, and concomitant therapy (both inhalers and nebulizers). Whereas peak flow and FEV_1_ did not differ statistically by device, patient-reported outcomes (symptoms and quality of life) showed greater improvements with nebulizer use, with some additional benefits reported by patients using concomitant therapy.

Brophy et al. ([42]; *n* = 25 with COPD) found no statistically significant difference between treatment with ipratropium bromide and albuterol via inhalers and nebulizers in measures of lung function, 6-min walk distance, breathlessness score, or quality-of-life score. Nonetheless, the majority of patients in this crossover trial (60%) reported a preference for nebulizers; no explanation for this finding was provided by the investigators.

#### Summary

For many years, spirometry remained the standard method for grading COPD severity [43]. However, as GOLD noted in their most recent (2020) report: “At an individual patient level, FEV_1_ is an unreliable marker of the severity of breathlessness, exercise limitation, and health status impairment [30].” The results of studies discussed in this section further suggest that objective measures of lung function, such as FEV_1_, are also unreliable markers for patient-reported preferences regarding inhalation device. For this reason, it is important to consider patients’ perspectives on how they perceive relief of dyspnea with various inhalation devices.

### Observational Studies

Observational studies, where the investigators observe patients in a non-controlled environment without manipulating their exposure, have both advantages and disadvantages relative to clinical trials. For example, potential confounding is less easily controlled in many observational studies, although longer follow-up times are usually possible. We identified one observational study that compared inhalers to nebulizers regarding hospital readmissions for AECOPD.

Loh et al. ([44]; *n* = 22 patients with sPIFR) conducted a retrospective analysis of patients hospitalized with AECOPD who were followed after release for hospital readmission, finding that all-cause and COPD 30- and 90-day readmission rates were significantly lower for those discharged with a nebulizer compared with DPI therapy, even though patients given nebulizers were older and had poorer measures of lung function at discharge (data not shown in tables). Drugs prescribed via nebulizer were similar to those prescribed via DPI (triple therapy with long-acting beta2-agonists (LABAs), long-acting muscarinic antagonists (LAMAs), and ICS).

#### Summary

Hospital readmissions are an understudied outcome in patients with COPD. Readmissions are costly, adversely affect quality of life and, if within 30 days, are currently subject to non-reimbursement under congressional mandate [45]. Therefore, if confirmed in future studies, the findings of Loh et al. [44] would have both clinical and economic implications [9].

### Patient Surveys of Inhalation Device Preference for Maintenance Therapy

Studies that compare inhaler devices and only consider objective measures of lung function do not appear to adequately capture meaningful differences in patients’ subjective disease and treatment experiences. Though not without methodological challenges, such as information accuracy and sample representativeness, surveys have the advantage of gathering subjective data directly from patients in their normal/home environment. However, few published studies have solicited feedback from patients with stable COPD regarding their symptoms, quality of life, satisfaction with their treatment, or inhaler device preference, after using both inhalers and nebulizers for maintenance treatment. We identified four such surveys of patients with stable COPD, including one that also surveyed a separate group of patient caregivers (Table 3).

**Table 3:** Surveys of patient-reported symptom control, quality of life, and device preference with nebulizers vs. inhalers

First author, year	Study type	Sample size	Study findings
Barta, 2002 [46]	Patient survey (via postal questionnaire)	82 with COPD	Approximately 75% of patients reported greater symptom relief with nebulizers than inhalers; 98% reported that the benefits of nebulized therapy outweighed any disadvantages; nebulized treatment at home helped patients feel comfortable and more in charge of their own symptom control; compliance was generally excellent
Sharafkhaneh, 2013 [47]	Telephone survey of randomly selected patients and caregivers	400 patients with COPD and 400 caregivers	Most patients and caregivers (~ 80%) preferred therapy with nebulizer vs. inhalers for controlling symptoms and improving quality of life
Dhand, 2018 [48]	Online survey using the Harris Poll Online panel	254 patients with COPD	54% of patients with COPD preferred nebulizers to other inhalation devices
Hanania, 2018 [49]	Web-based, descriptive, cross-sectional US-based survey	499 with self-reported COPD	Most (35%) patients reported no device preference, whereas 33% preferred pMDIs, 12% preferred nebulizers, 10% preferred SMIs, and 9% preferred DPIs. Patients with more severe symptoms (mMRC score ≥ 2) were most likely to report using a nebulizer

Barta et al. ([46]; *n* = 57 with COPD) sent questionnaires by mail to patients receiving home nebulizer treatment at their university hospital in London, England. Approximately 75% of patients reported greater symptom relief with nebulizers than inhalers; 98% reported that the benefits of nebulized therapy outweighed any disadvantages. Patients further reported that nebulized therapy at home helped them feel more in charge of their symptom control.

Sharafkhaneh et al. ([47]; *n* = 400 with COPD and 400 caregivers) used random digit dialing to interview patients and a separate group of caregivers from a national (USA) commercially available sample of self-reported COPD households. Based on their personal experiences, 80% of patients and caregivers reported that using a nebulizer was better than using only an inhaler, with improved symptom relief and quality of life. A large majority of patients and caregivers reported that the benefits of therapy with nebulizers outweighed any difficulty or inconvenience.

Dhand and coinvestigators ([48]; *n* = 254 with COPD) conducted an online survey using the Harris Poll Online panel, a US-based, voluntary, anonymized, survey site that prevented duplicate entries. Among subjects who had ever used them, nebulizers were preferred by a majority (54%) over other inhalation devices.

Hanania et al. ([49]; *n* = 499 patients with COPD) conducted a web-based, descriptive, cross-sectional US-based survey of individuals with self-reported COPD. Most (35%) patients reported no device preference, whereas 33% preferred pMDIs, 12% preferred nebulizers, 10% preferred SMIs, and 9% preferred DPIs. It was not reported what factors, such as “effectiveness” or “convenience,” influenced device choice, or to what extent patients had experience being treated with more than one device. A large majority (72%) of patients in this survey reported current use of nebulizers. Patients with more severe symptoms (mMRC score ≥ 2) were more likely to report using a nebulizer (49%) than a DPI (39%), pMDI (38%), or SMI (36%). A substantially greater percentage of patients who used nebulizers (82%) were “very confident about medication delivery” compared with patients who used DPIs (51%), pMDIs (57%), or SMIs (62%).

#### Summary

The results of these four surveys are consistent with those of clinical trials (discussed above) that considered patient-reported outcomes. Overall, these studies provide evidence refuting the blanket conclusion that nebulizers and inhalers are equally effective, at least from patients’ perspectives. The results of these studies support the opinion that nebulizer use is a viable option for many patients with stable COPD and certain clinical characteristics. We defined those characteristics in an earlier review [16].

## Discussion

Based on our review of consensus statements, clinical trials, observational studies, and patient surveys, there are two widespread assertions in the COPD community that may compromise patients’ treatment results and overall satisfaction with their treatment: (1) inhalers and nebulizers are equally effective when used appropriately, and (2) the majority of patients can be trained to use their inhalers appropriately. The extent to which the 2005 review by Dolovich et al. [31] has promulgated the assertion regarding inhaler-nebulizer equivalence is unknown, but the many times the work has been cited in the COPD literature suggest that it has had a considerable influence on device selection for inhaled medications. That review addressed studies of device–device comparisons that examined single-dose strengths of beta-agonist bronchodilators near the plateau of the dose–response curve, making it difficult to differentiate effects between devices. Furthermore, those studies mainly focused on objective measures of lung function, which do not appear to correlate well with patient-reported dyspnea symptoms or quality-of-life measures [30]. The evidence comparing inhalers versus nebulizers was especially weak, being based on two short-term studies that included a total of less than 70 patients with COPD. As discussed earlier, the results of studies that examined patient-reported outcomes do not tend to support the equivalence of bronchodilators given by inhalers and nebulizers.

The assertion that the majority of patients can be trained to use their inhalers appropriately is manifested in consensus group statements, discussed above, that generally recommend inhalers for patients with stable COPD, either without qualification or while at the same time noting that some patients may benefit from nebulizers when inhaler technique is compromised. One problem with this assertion is that it has been established by many investigators that an overwhelming majority of patients with COPD do not receive optimal relief from disabling symptoms because they do not use their inhalers in an optimal manner [8, 37, 38]. Regarding training, there is evidence that even extensive training may not largely mitigate patients’ misuse of inhalers. For example, in the survey conducted by Hanania et al. [49], 79% of patients with COPD reported at least one physical or cognitive impairment that could limit their ability to correctly manipulate an inhaler device, including arthritis, poor eyesight, poor hearing, memory problems, tremor, difficulty with fine motor activities, depression, or anxiety, and more than half of the respondents had multiple limitations. Consequently, even assuming inhaler–nebulizer equivalence with perfect use, the majority of patients with COPD may not achieve optimal benefits from inhalers due to co-morbid physical and cognitive limitations that cannot be improved by device training alone. Long-term pragmatic clinical trials that compare the effects of long-acting drugs via nebulizers versus inhalers in patients with stable COPD, while also comparing typical versus extensive device use training, may help to clarify this issue.

Patients and physicians generally have not considered the type of inhalation device or the proper technique of use to be important considerations in treatment choice [49], whereas GOLD guidelines have increasingly noted their importance [30]. Perhaps as a consequence, there has been little change in the high estimates of inhaler misuse over the past few decades [50]. Based on these trends, and the evidence reviewed here, assertions of general inhaler–nebulizer equivalence do not seem warranted or prudent. Rather, it seems prudent to recommend that patients and physicians pay greater attention to device selection, to the device use training regimen necessary to achieve and maintain proficiency, and to each patient’s potential physical and cognitive barriers to the success of that training.

Nebulizers are not free of potential problems with their use or in achieving adequate technique. For example, basic nebulizer inhalation technique, such as sitting in an upright position during therapy, may not be practiced by all patients [51]. Another problem associated with nebulizers is their lack of portability; an electrical or compressed gas source is needed for operation. When used in the home, nebulizer performance depends on the choice of an appropriate compressor [52, 53], and some nebulizer manufacturers specify the compatible compressors for optimum performance. Poor efficiency, residual volume of 1–3 ml that cannot be nebulized, continuous aerosol generation with loss of aerosol to the environment that poses a health risk, inconveniently long treatment time, need for equipment setup and cleaning, increase in the concentration of the solution and decrease in its temperature during nebulizer operation, limited access to accessories, the use of damaged parts, and patients engaging in self-repairs, are other problems associated with nebulizer use [51]. Moreover, shear forces generated during jet nebulization may inactivate or denature the active drug or agent. With continuous operation of a jet nebulizer, a significant amount of aerosol is wasted during exhalation, and several adaptations have been made in jet nebulizers to minimize this drug wastage. Nebulizers could also become contaminated with oral secretions if they are not cleaned properly [54]. However, with the recent evolution in nebulizer technology, some of the drawbacks of nebulizers, such as their lack of portability, difficulty with cleaning, and the longer duration for treatments, have been mitigated. Nebulizers can now be used with portable compressors that fit in a vehicle’s cigarette lighter, have the ability to deliver the treatment in 5–6 min, and can be cleaned in a dishwasher. Another limitation of nebulizers was the lack of availability of LAMAs in solution. The approval of glycopyrrolate (Lonhala, Sunovion) in 2018 and revefenacin (Yupelri, Mylan/Theravance) in 2019 has overcome this limitation. Whereas combinations of LABAs and LAMAs currently are not commercially available for nebulization, a formoterol (LABA) solution could be mixed with revefenacin (LAMA) solution without loss of efficacy [55].

The biological mechanisms that might underlie the differences in response between nebulizers and inhalers are unknown, but it is possible that with several slow inhalations over time, aerosolized medications delivered by nebulizer have more peripheral versus central deposition compared with inhalers [56–58]. Conceivably, the longer duration of inhaling bronchodilators by nebulization leads to more effective reduction in resting and dynamic lung hyperinflation, with consequent improvements in exercise tolerance and perceived breathlessness during exertion, even if nebulizers and inhalers still produce similar effects on FEV_1_ [59]. In any case, as detailed above, patients appear to sense a difference in response to medication delivered by nebulizer compared with inhalers.

## Conclusions

In conclusion, current guidelines for treatment of patients with COPD recommend bronchodilator delivery with nebulizers for patients experiencing acute breathlessness. In patients with stable disease, inhalers are generally recommended for maintenance therapy, and the use of nebulizers in this setting is often discouraged. In this article, we reviewed the weak scientific evidence that forms the basis for the latter recommendation. Recent investigations, especially those that include patient perceptions as an outcome measure, do not support the equivalence of bronchodilator therapy with nebulizers and inhalers. Indeed, if patients with stable COPD experience greater symptomatic benefit with nebulizers, then withholding nebulizer therapy from those patients may be denying them the ability to better control their symptoms, reduce acute exacerbations, and enhance their quality of life. We recommend well-designed comparative efficacy and safety trials with LABA/LAMA combinations, with or without ICS, administered by inhalers versus nebulizers to evaluate the role of nebulizers for maintenance therapy in patients with stable COPD.
